# The Impact of Thyroiditis on the Immune Profile in Young Patients with Uncomplicated Type 1 Diabetes

**DOI:** 10.3390/ijms25179721

**Published:** 2024-09-08

**Authors:** Jolanta Neubauer-Geryk, Małgorzata Myśliwiec, Katarzyna Zorena, Leszek Bieniaszewski

**Affiliations:** 1 Clinical Physiology Unit, Medical Simulation Centre, Medical University of Gdańsk, 80-210 Gdańsk, Poland; lbien@gumed.edu.pl; 2 Department of Pediatrics, Diabetology and Endocrinology, Medical University of Gdańsk, 80-211 Gdańsk, Poland; malgorzata.mysliwiec@gumed.edu.pl; 3 Department of Immunobiology and Environment Microbiology, Medical University of Gdańsk, 80-211 Gdańsk, Poland; kzorena@gumed.edu.pl

**Keywords:** angiogenin, proangiogenic cytokines, anti-inflammatory cytokines, proinflammatory cytokines, type 1 diabetes mellitus, Hashimoto thyroiditis, children

## Abstract

Autoimmune thyroid disease (AIT) is the most frequently linked autoimmune condition to type 1 diabetes (T1D). The analysis of immune profiles could provide valuable insights into the study of these diseases. This knowledge could play a crucial role in understanding the relationship between immune profiles and microcirculation structures and functions. The present study aimed to test the hypothesis that cytokine levels in T1D patients without and those with comorbid Hashimoto’s disease differ significantly. The total study group (total T1D) consisted of 62 diabetic young patients: 43 T1D and 19 T1D + AIT matched for age, age at onset, and duration of diabetes. The control group consisted of 32 healthy young subjects. The levels of cytokines (including TNF-α, IL-35, IL-4, IL-10, IL-18, IL-12, VEGF, and angiogenin) were quantified throughout this investigation. A comparative assessment of the cytokines profiles between the control group and total T1D revealed a statistically significant elevation in the levels of IL-4, TNF-α, IL-18, VEGF, and angiogenin, accompanied by a notable decline in IL-10. However, IL-35 and IL-12 exhibited comparable levels between the two groups. A comparison of cytokine levels between T1D + AIT and T1D groups revealed that only angiogenin levels were statistically significantly higher in T1D + AIT. The results of our study indicated that the alterations in cytokine levels associated with AIT did not correspond to the observed changes in T1D-related outcomes. The sole notable observation was the elevation of angiogenin expression, an angiogenic factor.

## 1. Introduction

Type 1 diabetes mellitus (T1D) is a chronic disease that affects around 1 in 300 children, and its prevalence has been steadily increasing over the last few decades [1,2].

The most commonly associated autoimmune condition with T1D is autoimmune thyroid disease (AIT) [3,4,5]. Research indicates that approximately one-third of individuals with T1D develop AIT within a few years from the onset. Together, type 1 diabetes and autoimmune thyroiditis are classified as a variant of autoimmune polyglandular syndrome type 3 (APS3v). It has been observed that in T1D patients, Hashimoto thyroiditis (HT) is relatively more frequent than Graves’ disease (GD) [5,6]. T1D and AIT are both caused by the infiltration of T cells and the production of autoantibodies against the islets of the pancreas and the thyroid gland, which leads to their dysfunction and destruction [2].

Poor glycemic control in diabetes is associated with heightened oxidative stress and increased protein glycation [7,8]. This has been linked to cytokine activity, including TNF-α, interleukin-6 (IL-6), and interleukin-12 (IL-12) family (IL-12, IL-27, IL-23, IL-35, and IL-39), pointing to their crucial role in the inflammatory response and T1D development [7,9,10,11,12,13,14,15,16,17].

IL-35 levels have also been found to have a significant negative correlation with high HbA_1c_ levels. This cytokine may be pivotal in reducing inflammation and improving insulin sensitivity in T1D [18].

On the other hand, IL-18 is positively correlated with HbA_1c_ levels [8,18], which suggests a potential association between IL-18 and worse glycemic control in these patients [8]. Research has shown that IL-4 protects against pancreatic β-cell loss in type 1 diabetes [19]. Additionally, IL-4, IL-10, and IL-13 cytokines have been suggested to activate the humoral immune response by stimulating B cells to release autoantibodies against islet cells and GAD molecules.

The pathogenesis of Hashimoto’s thyroiditis (HT) is considered to be multifactorial and remains incompletely understood. Several factors have been demonstrated to affect the risk of developing HT, including infectious agents, radiation, selenium, the presence of various comorbidities (e.g., diabetes, allergic rhinitis), and differentially expressed genes [5,20,21,22]. Genetic predisposition encompasses MHC genes (e.g., HLA-DR3) and genes that regulate the immune response, including the IL-7R (interleukin-7 receptor) gene. The cellular and molecular characteristics of thyroid autoimmunity in Hashimoto’s thyroiditis (HT) were meticulously examined by Luty et al. [23].

An imbalance in the Th1/Th2 cell ratio, with a predominance of Th1 cells, results in a complex dysfunction of the interplay between cells and cytokines. This leads to the destruction of thyroid cells, the release of cytokines such as IL-4, IL-5, IL-10, IL-6, and TNF-α, and the enhancement of the autoimmune process [24,25,26,27,28,29]. Additionally, there is an increase in the production of IL-1β, TGF-β, IFN-γ, IL-8, IL-23, and proinflammatory chemokines, which serves to enhance the autoimmune process. Additionally, research has demonstrated that patients with HT exhibit elevated expression of IL-22 and IL-17 [29,30,31]. IL-23/IL-17 has been linked to the pathogenesis and severity of HT, while augmented secretion of IL-23 may serve as a biomarker for the progression and monitoring of HT [32].

Some studies have demonstrated that vascular endothelial growth factor (VEGF) and angiogenin also play an important role in the clinical course of T1D and AIT.

VEGF has been demonstrated to stimulate the proliferation, migration, and vasopermeability of vascular endothelial cells in a range of tissues and cells. The available evidence suggests that serum levels of VEGF are associated with the clinical activity of some diseases, including tumor growth, coronary artery atherosclerosis, Kawasaki disease, rheumatoid-arthritis-associated synovial tissue neovascularization, and diabetic microangiopathy [33,34,35,36,37,38]. It has been demonstrated that VEGF produced by thyroid epithelial cells plays a pivotal role in intrathyroidal angiogenesis in patients with Graves’ disease and hypothyroid goitrous Hashimoto’s thyroiditis. The observed reductions in serum thyrotropin receptor antibody (TRAb) and thyroid stimulating hormone (TSH) levels appear to contribute to the observed decreases in serum VEGF levels, intrathyroidal vascular area, and thyroid volume [39]. However, a study by Figueroa-Vega et al. found no increase in serum VEGF levels in patients with GD or HT compared to healthy controls [40]. Similarly, a study by Semeran et al. [34] found no difference in plasma VEGF levels between diabetic and healthy children. The potential changes in serum VEGF levels in diabetes and HT are still under discussion.

Angiogenin is an anti-inflammatory cytokine that has been demonstrated to stimulate the activity, proliferation, and migration of endothelial cells during the early phases of angiogenesis [41]. Additionally, it has demonstrated anti-inflammatory and neuroprotective effects [41,42].

Angiogenin exerts its vasodilatory effect by NOS (nitric oxide synthase) activation and influences the formation of prostacyclin and NO (nitric oxide) [43,44]. Furthermore, it is involved in angiogenic processes under both physiological and pathological conditions, wound healing, tissue repair, and tumor growth [41]. Reduced serum levels in diabetic patients may contribute to impaired angiogenesis, especially in long-term diabetics [45,46]. In young patients with type 1 diabetes, increased microangiopathic complications in adolescence were shown to be associated with significantly increased angiogenin levels, whereas maintaining tight glycemic control led to decreased levels [47].

The clinical progression of autoimmune disorders is susceptible to a complex interplay of genetic, epigenetic, and environmental factors, which can exert differential effects on distinct organs within a single patient. The characterization of the immune profile may prove invaluable in the study of these diseases. This knowledge may prove instrumental in the analysis of correlations between immune profile and microcirculation structure and function.

The hypothesis we sought to test in the present study was whether the cytokine levels in patients with type 1 diabetes alone and a group with comorbid Hashimoto’s disease differed significantly from each other.

## 2. Results

### 2.1. Characteristics of Studied Subgroups

The study group consisted of 43 pediatric patients with type 1 diabetes and 19 with type 1 diabetes and coexisting autoimmune thyroiditis (T1D + AIT), matched for age and gender (Table 1). The patients with T1D and T1D + AIT were also matched for age at onset of T1D and diabetes duration. A control group of 32 healthy subjects of similar gender distribution was also included. The control subjects were significantly younger and had a lower BMI than the diabetic group. The study found no significant difference in the distribution of puberty stage according to the Tanner scale between the T1D and T1D + AIT groups (*p* = 0.98).

The diabetic groups did not differ significantly concerning patient BMI, current HbA_1c_, duration of insulin pump use or insulin dose, or the number of mild and severe hypoglycemia episodes (Table 1). However, the subgroups differed in treatment with the pump. Patients with Hashimoto’s disease used an insulin pump for a longer period compared to patients who only had diabetes.

### 2.2. Laboratory Examination

The total study group of patients with diabetes had significantly higher levels of total cholesterol and LDL fractions as well as significantly lower levels of HDL cholesterol compared to the control group. There was no significant difference in triglyceride levels between the two groups. Furthermore, there was no statistically significant difference between the diabetic and control groups in C-reactive protein levels. However, there was a borderline significant difference in creatinine concentration (*p* = 0.047) (Table 2).

The studied subgroups of diabetic patients did not differ regarding creatinine and lipid levels (Table 2). TSH levels differed significantly between subgroups. The T1D + AIT subgroup had significantly higher TSH levels. However, fT4 levels were not significantly different between the T1D and T1D + AIT groups (Table 2).

The levels of total cholesterol, LDL, and HDL fractions, as well as triglycerides, were found to be similar in patients with T1D and T1D + AIT. Furthermore, there were no statistically significant differences in the levels of creatinine and C-reactive protein between the study subgroups (Table 2).

### 2.3. Cytokines Examination

A comparison of the levels of anti-inflammatory cytokines between the control group and the total diabetic group revealed a significant elevation in IL-4 and a decrease in IL-10 in total D1M, whereas IL-35 remained unaltered. Regarding proinflammatory cytokines, it was observed that TNF-α and IL-18 were significantly elevated in the total T1D group, while IL-12 remained comparable. A comparative analysis of VEGF and angiogenin levels demonstrated a clear elevation in these cytokines in the whole diabetic group compared to the control group (Table 3).

**Figure 1:**
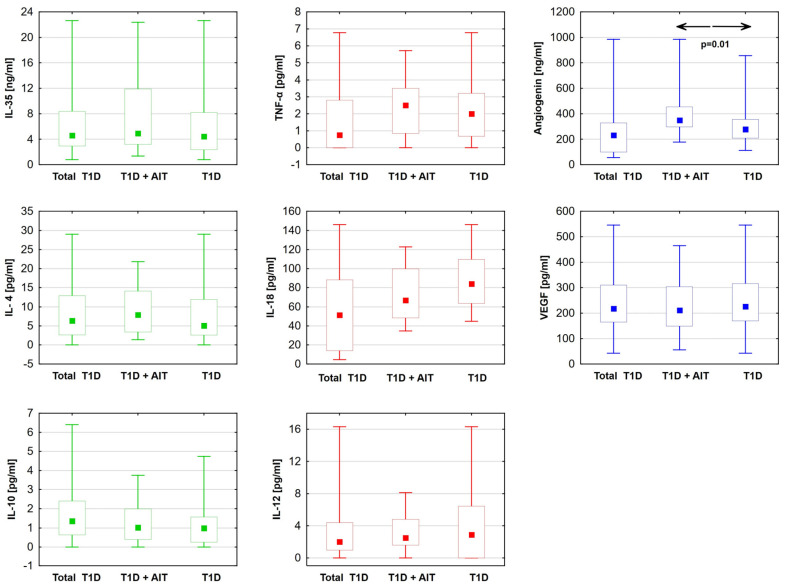
Differences in cytokine levels: anti-inflammatory (green). Proinflammatory (red) and angiogenic factors (blue) between diabetic patients with Hashimoto disease (T1D + AIT) and without (T1D). Data are presented in box plots (median and range). The value of *p* < 0.05 was regarded as statistically significant. ←→—between groups T1D + AIT and T1D; TNF-α—tumor necrosis factor; IL-35—interleukin 35; IL-4—interleukin 4; IL-10—interleukin 10; IL-12—interleukin 12; IL-18—interleukin 18; T1D—diabetes mellitus; AIT—autoimmune thyroiditis.

The levels of anti-inflammatory (IL-35, IL-10, IL-4) and proinflammatory (TNF-α, IL-12, IL-18) cytokines were statistically comparable between the type 1 diabetes patients with Hashimoto’s disease and those without. Similarly, VEGF levels were comparable across the study groups. However, the angiogenin level was found to be statistically significantly higher in the group of patients with both diabetes and Hashimoto’s disease (Table 3) (Figure 1).

## 3. Discussion

The activity of the immune processes, expressed by the balance of cytokines, reflects the common background and predisposing factors shown in several populations. The last decades of studying the clinical course of type 1 diabetes (T1D) revealed complex interplay and dynamics between elements of the immune profile.

The presented analysis of the levels of selected cytokines in patients with diabetes mellitus, with and without Hashimoto’s disease, revealed that only angiogenin levels were significantly elevated in the group with Hashimoto’s disease. In the case of other studied cytokines (IL-4, IL-10, IL-12, IL-18, IL-35, TNF-α, and VEGF), we found comparable levels in both groups.

The findings of our study indicated that the levels of angiogenin were also notably elevated in young patients with diabetes when compared to the control group (Table 3). Some studies investigated the associations between serum angiogenin levels in type 1 diabetes mellitus [48,49,50,51]. Researchers have also obtained comparable results in groups of children and adolescents with type 1 diabetes. They reported elevated levels of angiogenin in young patients with type 1 diabetes, irrespective of the degree of microvascular complications [47,51]. In middle-aged patients with type 1 diabetes, the angiogenin level was found to be lower than in controls. Moreover, published results showed ambiguous relations between the presence of diabetic complications and angiogenin level complications [47,51]. Our present findings indicate that the level of angiogenin in diabetic patients with HT is significantly higher in comparison to the group without HT. To the best of our knowledge, this is the first study to demonstrate angiogenin levels in young patients with T1D who have not yet developed microangiopathic complications but who also have comorbid Hashimoto’s disease.

The other studied angiogenic factor, namely, VEGF, did not differ between diabetic groups. The group of diabetic patients with Hashimoto’s disease exhibited significantly elevated TSH levels compared to the group without Hashimoto’s disease. However, it is important to note that all patients were in the euthyroid stage. The literature has shown inconsistent findings related to the analysis of VEGF levels in individuals with HT. It has been demonstrated that VEGF levels are markedly elevated in untreated HT patients with hypothyroidism in comparison to controls [39,52]. Additionally, some researchers showed that VEGF levels were the same [39], while others reported a lowering of plasma VEGF levels in treated HT patients with euthyroidism in comparison to controls [53].

Evidence suggests that VEGF plays a critical role in the development of diabetes-related complications [34,35]. In individuals with type 1 diabetes, elevated glucose levels precipitate the upregulation of VEGF, which in turn stimulates neovascularization and enhances the thickness of endothelial basement membranes and vascular permeability. A study by Chiarelli’s group [35], involving young patients with onset of diabetes before age 12 and a disease duration of at least 2 years, demonstrated increased serum VEGF levels in prepubertal and pubertal children with diabetes. There is a notable correlation between glycemia levels and serum VEGF concentrations [35]. The findings of our study confirm elevated levels of VEGF in young patients diagnosed with type 1 diabetes compared to healthy controls (Table 3).

We found no significant differences in the levels of studied anti-inflammatory cytokines in subgroups of diabetic patients with and without HT. For the overall T1D population compared to controls, we found relationships that are generally consistent with the limited data found in the literature.

Numerous studies have demonstrated that IL-35 plays a vital role in enhancing glycemic control and defending against T1D by regulating macrophage polarization and T-cell-associated cytokine ratios [54,55]. Elevated levels of IL-35 contribute to the prolonged preservation of pancreatic β-cell secretory function, thereby reducing the risk of hypoglycemia and diabetic complications [56]. IL-35 has been found to elevate inflammatory cytokine levels [17] during acute and chronic inflammatory processes such as atherosclerosis [14,55,57,58,59]. The result we observed is likely attributable to the relatively effective management of diabetes, which has resulted in the absence or reduction in diabetic microangiopathic complications. Reduced IL-35 levels have been shown to be associated with autoimmune hypothyroidism and its severity by Yilmaz et al. [60]. According to the authors, IL-35 has a protective effect against autoimmune thyroid tissue destruction.

Other anti-inflammatory cytokines, IL-4 and IL-10, have been proposed to activate the humoral immune response by stimulating B cells to release autoantibodies against islet cells and GAD molecules. The concentration of IL-4 was reported to be markedly higher in children with T1D in comparison to healthy controls [18,61] and in the HT group [62]. Our data confirmed the impact of T1D but not HT on this cytokine level. Our results demonstrated that the IL-10 levels were markedly diminished in the diabetic cohort relative to controls. Furthermore, no statistically significant differences were observed in IL-10 levels between individuals with and without HT. This finding is in line with the results of the study conducted by Myśliwiec and colleagues, which involved children diagnosed with type 1 diabetes, and revealed a notable decline in the serum levels of IL-10 [63]. Other researchers reported elevated IL-10 in patients with T1D as compared to healthy individuals [7,18,61]. Elevated IL-10 levels were also found in individuals with Hashimoto’s thyroiditis compared to healthy controls [64,65]. Elevated IL-10 levels were found to inhibit experimental autoimmune thyroiditis [66].

This study showed no statistically significant differences in the concentrations of the proinflammatory cytokines studied in the two subgroups of diabetic patients with and without HT. When the data were analyzed for the whole T1D group in comparison to the control group, the results showed findings that were in line with the limited existing literature on the matter.

TNF-α is a cytokine that has been demonstrated to play an essential role in the inflammatory process [11,13,67]. TNF-α has been linked to the development of complications related to diabetes [68,69]. Indeed, there is evidence that TNF-α is detectable in the serum of children and young adults with type 1 diabetes who also have nonproliferative diabetic retinopathy. Our findings demonstrated that the concentration of TNF-α was markedly elevated in the cohort of patients with type 1 diabetes when compared to the control group. These findings are consistent with the conclusions of other researchers in the field [70].

Data on TNF-α levels in thyroiditis are inconclusive. Patients with hypothyroidism or hyperthyroidism have been shown to have high plasma TNF-α levels. Treatment of hyperthyroidism resulted in a significant reduction in previously elevated TNF-α levels. Such reduction was not observed when thyroid function was normalized in patients with hypothyroidism [71]. However, Tayde et al. showed that TNF-α was significantly higher in patients with autoimmune hypothyroidism as compared to controls. These levels decreased with treatment but did not return to the baseline levels seen in the control group [72].

IL-18 is known to enhance natural killer (NK) cell and macrophage activity [64,65,66,73]. As shown in our study and previously by Al-Dubayee et al. [18], significantly elevated levels of IL-18 were observed in the diabetic group compared to controls. IL-18 has demonstrated a positive correlation with HbA_1c_ levels in type 1 diabetes, suggesting a potential association between IL-18 and glycemic control in these patients [8,18]. IL-18 levels were found to be elevated in diabetic nephropathy [74] and have been linked to obesity [75], insulin resistance [75], hypertension [76], and dyslipidemia [77,78].

We found no significant differences in IL-12 levels between groups of diabetic patients with and without Hashimoto’s disease, nor between diabetic patients and healthy controls. Such a difference was reported by Siddiq et al. [64]. The discrepancies observed were due to the fact that their study [64] excluded patients with T1D from the study cohort.

## 4. Limitation of the Study

Due to financial constraints, the set of cytokines analyzed was limited during the study’s planning phase. This fact may be considered a potential limitation of our study.

We are aware of the fact that the control group was not fully matched to the entire cohort of patients with type 1 diabetes and exhibited a significantly younger age and a lower BMI, and in contrast to the patients with diabetes, the control group did not undergo an assessment of their pubertal stage. A review of the controls’ medical history and physical symptoms did not suggest any abnormalities in thyroid function. Therefore, the lack of data on thyroid hormones does not represent a significant limitation of the study.

Our study was realized in rather small groups. A larger number of subjects would allow a better understanding of the age-related changes in the immune profile.

## 5. Materials and Methods

### 5.1. The Study Design and Population

The study group consisted of 62 patients with type 1 diabetes (33 girls and 29 boys, with an average age of 15.4 ± 2.3 years) (Table 1). We included patients with a minimum diabetes duration of 1.2 years who met the type 1 diabetes diagnostic criteria according to the International Society of Children and Adolescent Diabetes [79]. Patients were recruited from the Department of Pediatrics, Diabetology, and Endocrinology at the Medical University of Gdańsk between 2014 and 2018. Based on medical history, physical examination, and biochemical analysis, none of the subjects had any form of microangiopathy, including retinopathy, nephropathy, or neuropathy.

Individuals with diabetic ketoacidosis at the time of enrollment, ongoing infection, uncontrolled celiac disease, and chronic kidney disease were not included in the study. Additionally, individuals with endocrine disorders other than Hashimoto’s disease and those who had experienced severe hypoglycemia in the previous month were also excluded.

Patients who had been prescribed statins were not included in the analysis. Severe hypoglycemia was defined as a blood glucose level below 54 mg/dL, requiring assistance from another person. This episode occurred within one year of the study, but not more than one month before the study. Mild hypoglycemia was considered as a drop in blood glucose levels within one month prior to the study without the need for assistance [80].

The control group comprised 32 healthy children and adolescents (19 girls and 13 boys, with an average age of 13.8 ± 3.4 years) with no history of chronic diseases and who were not taking any medications (Table 1). In a similar manner to the diabetic patients, any infection was also considered an exclusion factor in the control group. They were recruited from schools or referred to the Diabetes Outpatient Clinic due to suspected endocrine disorders, which were eventually ruled out.

This study was conducted following the ethical standards of the Ethics Committee of the Medical University of Gdańsk and the 1964 Declaration of Helsinki as amended or comparable ethical standards. The Ethics Committee of the Medical University of Gdańsk approved the study protocol (NKBBN/277/2014; NKBBN/277-512/2016), and informed consent was obtained from all participants. Parents consented and participated in the study with their children.

The blood tests were administered to fasting participants between 7 and 9 a.m. The serum was separated from venous blood within 30 min and stored frozen at −80 °C for up to three months prior to analysis. All measurements were performed on the same sample.

### 5.2. Laboratory Analyses

Blood samples were collected to assess the levels of HbA_1c_, CRP, total cholesterol, high-density lipoprotein cholesterol (HDL-C), low-density lipoprotein cholesterol (LDL-C), triglycerides (TG), TSH, fT4, serum creatinine, and cytokines level.

HbA_1c_ was measured by an immunoturbidometric method using the Unimate 3 set (Hoffmann-La Roche AG, Basel, Switzerland). An enzymatic test (Roche Diagnostics GmbH, Mannheim, Germany) was used to measure fasting glucose, The level of C-reactive protein was measured by an immunochemical system (Beckman Instr, Inc., Galway, Ireland). The levels of total cholesterol, HDL and LDL cholesterol, and triglycerides were measured using Cormay enzymatic kits (Cormay, Lublin, Poland). Serum creatinine levels were measured using the CREA assay system (Boehringer Mannheim GmbH, Mannheim, Germany).

Serum concentrations of IL-4, IL-10, IL-18, IL-12, IL-35, and TNF- α were measured by enzyme-linked immunosorbent assay (ELISA) according to established manufacturer protocols. The serum levels of IL-4, IL-10, and IL-18 were quantified by ELISA using the R&D Systems Quantikine High Sensitivity Human kit (Minneapolis, MI, USA), according to the manufacturer’s protocol. The minimum detectable concentrations were established by the manufacturer to be 10, 0.5, and 5.15 pg/mL, respectively. The intra- and interassay precision of the assays for IL-4, IL-10, and IL-18 were determined, respectively, to be 2.7%, 6.6%, and 2.9%, and 7.4%, 8.1%, and 8.4%. Serum concentrations of TNF-α and IL-12 were quantified by ELISA (Quantikine High Sensitivity Human from R&D Systems, Minneapolis, MI, USA) in accordance with the manufacturer’s instructions.

The intra- and interassay coefficients of variation (CV) for TNF-α and IL-12 were found to be 6.2%, 2.6%, 2.5%, and 7.6%, respectively. Human IL-35 was quantified by ELISA (Thermo Fisher Scientific, Inc., Waltham, MA, USA). The interassay CV was found to be less than 10%, and the intra-assay CV was also found to be less than 10%. The sensitivity of the assay was 9.38 pg/mL. The absorbance of IL-4, IL-10, IL-18, IL-35, IL-12, and TNF-α was determined by reading at 450 nm on a CHROMATE 4300 automated plate reader (Awareness Technology, Inc., Palm City, FL, USA). The reference curve was generated in accordance with the manufacturer’s recommendations.

Serum VEGF (VEGF A) was quantified by ELISA using the Quantikine High Sensitivity Human kit (R&D System, Minneapolis, MN, USA) in accordance with the manufacturer’s instructions (catalog #: DVE00). The manufacturer has established a minimum detectable concentration of 5.0 pg/mL. The CV for the intra-assay was 5.1% and for the inter-assay, it was 6.2%.

The serum angiogenin level was quantified by an enzyme-linked immunosorbent assay (ELISA) using a commercially available kit (Quantikine High Sensitivity Human by R&D System, Minneapolis, MN, USA). The minimum detectable dose and the intra- and interassay CV of the assay were, respectively, 0.6 ng/mL and 3.0% and 8%.

### 5.3. Statistical Analysis

The statistical analysis was performed using STATISTICA version 13.1 from StatSoft Inc. Tulsa, OK, USA, under license CSM GUMed JPZP5077539317AR-H. The Shapiro–Wilk test was utilized to assess the distributions of continuous variables. Results were reported as either the median and range or the mean and standard deviation. In the absence of a normal distribution of the study variables, their values were compared using the nonparametric Mann–Whitney U-test. The chi^2^ test with Yates correction was used where appropriate. The χ^2^ test was used to compare the proportions of sex, stage of sexual maturity according to the Tanner scale, and the number of mild and severe hypoglycemic episodes. A *p*-level of <0.05 was considered to indicate statistical significance.

## 6. Conclusions

Our study showed that the changes in cytokines levels related to presence of immune thyroid inflammation is not reflected in the picture related to diabetes itself. The only significant observation that was found is an increase in the expression of the angiogenic factor, angiogenin.

The results of recent years have demonstrated the significance of several factors that were not included in the original study. It would be beneficial to conduct a prospective study examining the dynamics of cytokine levels in patients with diabetes and other concomitant autoimmune disorders, categorized by subgroups with varying onset and duration of diabetes, and a controlled stage of maturity. A comparison of the obtained results with data from healthy individuals will provide a comprehensive understanding of the immunological changes.

## Figures and Tables

**Table 1:** Characteristics and comparison of the study groups: a whole group of patients with diabetes (Total T1D) and diabetic patients with Hashimoto disease (T1D + AIT) or without (T1D) and controls (C).

Characteristics	Healthy Control C n = 32	Diabetic Patients			*p* for between-Group Comparisons	
			Subgroups		C vs. Total T1D	T1D vs. T1D + AIT
		Total T1D n = 62	T1D n = 43	T1D + AIT n = 19		
Males. n (%)	13 (40.6)	29 (46.8)	22 (51.2)	7 (36.8)	0.57	0.44
BMI [kg/m^2^]	18.1 (14–26)	20.4 (14.51–29.7)	20 (14.5–30)	21 (16.1–27)	0.008	0.11
Age [years]	13.5 (7–20)/ 13.8 ± 3.4	15.7 (8.44–18)/ 15.4 ± 2.3	16 (8.4–18)/ 15 ± 2.3	15 (11.1–18)/ 15 ± 2.2	0.01	0.98
Onset of diabetes [age]	na	8.3 (1.15–13.6) 7.6 ± 3.6	9 (1.8–14)/ 8 ± 3.8	7 (1.2–12)/ 7 ± 3.1	na	0.11
Diabetes duration [years]	na	7.5 (1.18–15.9) 7.8±3.9	7 (1.2–16)/ 7± 4	9 (2.7–14)/ 9 ± 3.4	na	0.13
Insulin dose units/24 h	na	45 (20–100)	45 (25–100)	47 (20–90)	na	0.50
Insulin dose units/kg	na	0.8 (0.4–1.4)	0.8 (0.5–1.3)	0.8 (0.4–1.4)	na	0.99
Treatment with pump [%]	na	62 (0–100)	49 (0–100)	83 (0–100)	na	0.03
HbA_1c_ current [%]	4.8 (4–6)	8 (5.9–13.4)	8 (6.2–13)	8 (5.9–13)	<0.001	0.94
Episodes of mild hypoglycemia [N/last month]	na	10 (0–30)	10 (0–30)	10 (1–20)	na	0.21
Episodes of severe hypoglycemia [N/last year]	na	0 (0–2)	0 (0–1)	0 (0–2)	na	0.07

Data are presented as median (range)/mean values ± SD. The value of *p* < 0.05 was regarded as statistically significant. na—not applicable.

**Table 2:** Results and comparison of laboratory results in studied groups: a whole group of patients with diabetes (Total T1D) and diabetic patients with Hashimoto disease (T1D + AIT) or without (T1D) and controls (C).

Characteristics	Healthy Control C n = 32	Diabetic Patients			*p* for between-Group Comparisons	
			Subgroups		C vs. Total T1D	T1D vs. T1D + AIT
		Total T1D n = 62	T1D n = 43	T1D + AIT n = 19		
Serum creatinine [mg/dL]	0.7 (0.45–0.99)	0.69 (0.45–0.95)	0.69 (0.45–0.95)	0.65 (0.5–0.86)	0.047	0.15
CRP [mg/dL]	0.33 (0.02–1.28)	0.49 (0.1–4.9)	0.41 (0.1–4.9)	0.54 (0.12–4.3)	0.08	0.61
Total cholesterol [mg/dL]	161 (108–198)	178.5 (125–288)	178 (125–288)	180 (129–270)	0.002	0.27
Cholesterol LDL [mg/dL]	59 (37–82)	106 (61–188)	106 (61–188)	106 (68–180)	<0.001	0.22
Cholesterol HDL [mg/dL]	70 (34–99)	55 (33–90)	52 (33–90)	60 (42–65)	0.003	0.18
Triglycerides [mg/dL]	78 (37–98)	74 (34–294)	72 (37–294)	83 (34–160)	0.71	0.92
TSH [mIU/L]	na	1.93 (0.57–5.1)	1.62 (0.62–4.0)	2.1 (0.57–5.1)	na	0.01
fT4 [pmol/L]	na	12.5 (9–15)	13 (9–15)	12.8 (9.3–15)	na	0.63

Data are presented as median (range). The value of *p* < 0.05 was regarded as statistically significant. na—not applicable.

**Table 3:** Results and comparison of cytokines level in studied groups: a whole group of patients with diabetes (Total T1D) and diabetic patients with Hashimoto disease (T1D + AIT) or without (T1D) and controls (C).

Characteristics	Healthy Control C n = 32	Diabetic Patients			*p* for between-Group Comparisons	
			Subgroups		C vs. Total T1D	T1D vs. T1D + AIT
		Total T1D n = 62	T1D n = 43	T1D + AIT n = 19		
anti-inflammatory cytokines						
IL-35 [ng/mL]	5.9 (1.2–16)	4.5 (0.8–22.7)	4 (0.8–23)	5 (1.4–22)	0.30	0.29
IL-4 [pg/mL]	2.5 (0–6)	6.3 (0–29)	5 (0–29)	8 (1.4–22)	<0.001	0.39
IL-10 [pg/mL]	2.3 (0–6)	1 (0–4.7)	1 (0–5)	1 (0–4)	<0.001	0.50
proinflammatory cytokines						
TNF-α [pg/mL]	0 (0–1)	2.2 (0–6.8)	2 (0–7)	3 (0–6)	<0.001	0.37
IL-12 [pg/mL]	1.7 (0.4–4)	2.7 (0–16.3)	3 (0–16)	3 (0–8)	0.081	0.97
IL-18 [pg/mL]	11.7 (4.7–19)	78.5 (34.6–146)	84 (44.8–146)	67 (35–123)	<0.001	0.16
angiogenic factors						
Angiogenin [ng/mL]	78 (56–272)	298.8 (112–985)	279 (112–856)	349 (179–985)	<0.001	0.01
VEGF [pg/mL]	109.7 (55.7–211)	218.1 (43.04–546.2)	226 (43–546)	212 (55.6–465)	<0.001	0.87

Data are presented as median (range). The value of *p* < 0.05 was regarded as statistically significant.

## Data Availability

The data presented in this study are available on request from the corresponding author.

## Abbreviations

AIT—autoimmune thyroiditis, APS3v- autoimmune polyglandular syndrome type 3 variant. anti-TPO—anti-thyroid peroxidase autoantibodies. HT—Hashimoto thyroiditis, HbA_1c_—glycated hemoglobin, VEGF—vascular endothelial growth factor, BMI—body mass index, T1D—diabetes mellitus, TSH—thyroid-stimulating hormone, fT4—free thyroxine, TNF-α—tumor necrosis factor, IL—interleukin, TGF-β—transforming growth factor beta, IFN—interferon gamma, CRP—C-reactive protein, LDL-cholesterol—low-density cholesterol, VLDL-cholesterol—very low-density cholesterol, HDL—high-density cholesterol, NOS—nitric oxide synthase, NO—nitric oxide.
